# Immunoproteomic analysis of the excretory-secretory products of *Trichinella pseudospiralis* adult worms and newborn larvae

**DOI:** 10.1186/s13071-017-2522-9

**Published:** 2017-11-21

**Authors:** Yang Wang, Xue Bai, Haichao Zhu, Xuelin Wang, Haining Shi, Bin Tang, Pascal Boireau, Xuepeng Cai, Xuenong Luo, Mingyuan Liu, Xiaolei Liu

**Affiliations:** 1 Key Laboratory of Zoonosis Research, Ministry of Education, Institute of Zoonosis/College of Veterinary Medicine/College of Basic Medical Science, Jilin University, Changchun, China; 2 Mucosal Immunology Laboratory, Pediatric Gastroenterology Unit, Massachusetts General Hospital East, Boston, USA; 3 ANSES, INRA, ENVA, Universite Paris Est, Laboratory for Animal Health, Maisons Alfort, Paris, France; 4 China Institute of Veterinary Drugs Control, Beijing, 100000 China; 5 State Key Laboratory of Veterinary Etiological Biology, Key Laboratory of Veterinary Parasitology of Gansu Province, Lanzhou Veterinary Research Institute, CAAS, Lanzhou, 730046 China; 6 Jiangsu Co-innovation Center for Prevention and Control of Important Animal Infectious Diseases and Zoonoses, Yangzhou, China

**Keywords:** *Trichinella pseudospiralis*, Excretory-secretory proteins, Immunoproteomics

## Abstract

**Background:**

The nematode *Trichinella pseudospiralis* is an intracellular parasite of mammalian skeletal muscle cells and exists in a non-encapsulated form. Previous studies demonstrated that *T. pseudospiralis* could induce a lower host inflammatory response. Excretory-secretory (ES) proteins as the most important products of host-parasite interaction may play the main functional role in alleviating host inflammation. However, the ES products of *T. pseudospiralis* early stage are still unknown. The identification of the ES products of the early stage facilitates the understanding of the molecular mechanisms of the immunomodulation and may help finding early diagnostic markers.

**Results:**

In this study, we used two-dimensional gel electrophoresis (2-DE)-based western blotting coupled with matrix-assisted laser desorption/ionization time of flight mass spectrometry (MALDI-TOF/TOF-MS/MS) to separate and identify the *T. pseudospiralis* adult worms ES products immunoreaction-positive proteins. In total, 400 protein spots were separated by 2-DE. Twenty-eight protein spots were successfully identified using the sera from infected pigs and were characterized to correlate with 12 different proteins of *T. pseudospiralis*, including adult-specific DNase II-10, poly-cysteine and histidine-tailed protein isoform 2, serine protease, serine/threonine-protein kinase ULK3, enolase, putative venom allergen 5, chymotrypsin-like elastase family member 1, uncharacterized protein, peptidase inhibitor 16, death-associated protein 1, deoxyribonuclease II superfamily and golgin-45. Bioinformatic analyses showed that the identified proteins have a wide diversity of molecular functions, especially deoxyribonuclease II (DNase II) activity and serine-type endopeptidase activity.

**Conclusions:**

Early candidate antigens from the ES proteins of *T. pseudospiralis* have been screened and identified. Our results suggest these proteins may play key roles during the *T. pseudospiralis* infection and suppress the host immune response. Further, they are the most likely antigen for early diagnosis and the development of a vaccine against the parasite.

## Background

Trichinellosis is an important food-borne parasitic zoonosis that infects humans and other mammals, with outbreaks in many parts of the world [1, 2]. Humans acquire the disease by consuming raw or undercooked meat of pigs and other animals containing the infective larvae of *Trichinella spiralis* [3]. Nine different species and three genotypes have been identified to date. Among them, *T. nativa*, *Trichinella T6*, *Trichinella T9*, *T. murrelli*, *Trichinella T8*, *T. britovi*, *T. patagoniensis*, *T. nelsoni* and *T. spiralis* are encapsulated in the host muscle tissues with the formation of collagen layer. *Trichinella pseudospiralis*, *T. papuae* and *T. zimbabwensis* do not induce formation of the collagen layer in the nurse cell [4]. *Trichinella spiralis* and *Trichinella pseudospiralis* are independent and typical species in the genus *Trichinella*. These two species are similar but differ in certain host responses, such as capsule morphology, gene expression, immunological responses and ES products.

After being ingested with infected muscle tissue, the muscle infective larvae (ML) are released and invade the small intestinal epithelium, where larvae complete four moults in 30–40 h and develop into adult worms. The female begins to release the newborn larvae (NBL) over a period of 5–10 days. The NBL penetrate the intestinal wall through the blood and lymphatic circulation into striated muscle, where they grow and form encapsulated and non-encapsulated forms [5]. *Trichinella pseudospiralis* has a worldwide distribution in Europe, Asia, North America and Australia. It has been detected to infect sylvatic predators such as pigs and rats [6, 7], lynx [8] and red foxes [9]. Moreover, this species can infect humans [10] and is the only species that infects birds [11].

*Trichinella spiralis* and *T. pseudospiralis* ES products are very similar but are not identical in cDNA sequence, molecular mass, antigenicity and peptide maps of ES products [12–15]. ES products are considered to be directly exposed to the host’s immune system, which induces the host immune responses. Consequently, ES products may play a crucial role in the invasion and development of *Trichinella* larvae [16, 17]. The ES products of *T. spiralis* include some functional proteins, such as heat shock proteins [18], endonucleases [19], proteinases [20], protein kinases [21, 22], proteinase inhibitors [23], DNA binding [24], and 5′-nucleotidase [25]. The ES products of *T. pseudospiralis* are likely involved in products that have been published, including gp 38, TppSP-1, 45 kDa antigen, TpSerP and 21 kDa ES [17]. The study of the ES products of *T. pseudospiralis* that modulate the host environment to allow parasite development and survival is of fundamental importance to identify the mechanisms leading to immunosuppression and relieving the host inflammatory response in *T. pseudospiralis*-infected host and may provide good markers for diagnosis and candidates for drug and vaccine development.

Recent advances in technology, such as western blotting, indirect immunofluorescence, enzyme-linked immunosorbent assay (ELISA) and proteomics have been utilized to identify the ES proteins of *Trichinella* spp*.* [26, 27]. Proteomics-based analyses involve the simultaneous separation, visualization and quantification of thousands of proteins. More importantly, the combination of proteomics with immunoblotting assays may discover more species-specific antigens than one-dimension analysis can. 2-DE and western blotting combined with MALDI-TOF/TOF-MS/MS are an effective approach for the high-resolution analysis and identification of complex groups of ES products.

## Methods

### Parasites and animals

*Trichinella pseudospiralis* preserved in Food-Borne Parasitology Laboratory of Key Laboratory for Zoonoses Research, Ministry of Education, Institute of Zoonoses, Jilin University were genotyped and proved by OIE Collaborating Center on Foodborne Parasites in Asian-Pacific Region in August 2014. *Trichinella pseudospiralis* (ISS13) ML were isolated by pepsin-HCl digestion from infected mice at 30 days. Adult worms and NBL were isolated from the infected mice intestines at 6 days post-infection (dpi) [28].

### Collection and preparation of *T. pseudospiralis*-infected animal sera

*Trichinella pseudospiralis*-infected pig sera were collected from 4 pigs orally infected with 10,000 worms/pig for 26 days. Uninfected sera from the same pigs before infection were collected as negative controls.

### Collection of *T. pseudospiralis* adult worms and NBL proteins sample preparation

The collected adult worms and NBL were washed several times with sterile PBS and then were cultured in pre-warmed RPMI-1640 supplemented with 100 U penicillin/ml and 100 μg streptomycin/ml. The adult worms and NBL were incubated at 5000 worms/ml for 20 h at 37 °C in 5% CO_2_. After incubation, the media containing the ES proteins were centrifuged at 1000× *rpm* at 4 °C for 5 min and the supernatant containing the ES products were filtered through a 0.2 μm membrane into ultrafiltration device. The ES products were centrifuged at 5000× *rpm* at 4 °C and were concentrated to 100 μl. The total protein concentration was determined by Bradford assay [29].

### Two-dimensional gel electrophoresis

Three replicates of the ES proteins samples were run in parallel on three immobilized pH gradient (IPG) strips. Normally, 100 μl of ES proteins were diluted to 360 μl in rehydration buffer and were loaded into the precast IPG strips (pH 4–7 NL, 17 cm, GE Healthcare, Fairfield, USA). ES proteins were gradient separated by isoelectric focusing (IEF) (GE ETTAN IPGPHOR3). The three IPG strips were rehydrated overnight for 12 h at 20 °C, followed by IEF under a running parameter (a gradient at 500 V and 1 h for 500 Vh, a gradient at 1000 V and 1 h for 1000 Vh, a gradient at 8000 V and 3 h for 24,000 Vh, a step and hold at 8000 V and 2.4 h for 19,200 Vh, and a step and hold at 500 V and 0.5 h for 250 Vh) to achieve a final level of approximately 45,000 Vh and 8 h (using a limit of 50 μA/strip). After IEF, the IEF strips were first equilibrated in an 8 ml reducing buffer (6 M urea, 2% sodium dodecyl sulfate (SDS), 50 mM Tris-HCl pH 8.8, 30% glycerol and 100 mM dithiothreitol) approximately 15 min, followed by an 8 ml alkylation buffer (6 M urea, 2% SDS, 50 mM Tris-HCl, pH 8.8, 30% glycerol and 250 mM iodoacetamide) for approximately 15 min. Then, the IPG strips were processed by the second-dimensional electrophoresis.

The equilibrated three IPG strips were loaded onto 12% SDS-PAGE gels by mixing 16 ml of 400 g/l acrylamide/bisacrylamide (29:1 by weight), 10 ml of 1.5 mol/l Tris-HCl (pH 8.8), 13.48 ml of distilled and deionized water, 100 μl of ammonia persulfate (Amersham, Fairfield, USA) and 20 μl of tetramethylethylenediamine (TEMED, Amersham). Next, 10 g/l low-molecular-weight agarose in SDS electrophoresis buffer was boiled to seal the equilibrated IPG strips to the top of the resolving gel. Gels were run at 2 w/gel for 60 min and then at a constant 17 w/gel until the dye front reached the bottom. The proteins of one gel were stained with Coomassie brilliant blue G-250 (Bio Basic, Amherst, USA) for 6 h. Images of the gels were captured using MICROTEK ScanMakeri800.

### Western blotting

The separated protein spots by 2-DE were transferred to a polyvinylidene fluoride (PVDF) membrane with a wet transfer cell (400 mA, 2.5 h). The PVDF membranes with the ES proteins were blocked with 5% skim milk in TBST (80 ml) for 1 h at room temperature. The TBST-blocked PVDF membrane was incubated (overnight, 4 °C) with *T. pseudospiralis*-infected swine pooled sera diluted 1:1000 in TBST. After completion of the incubation, the PVDF membrane was washed with the TBST solution (5 min × 3), and then the membrane was again incubated with the horseradish peroxidase-conjugated goat anti-mouse IgG (BioRad, Hercules, USA) (1:8000, 1 h, room temperature). The membrane was washed with TBST solution (5 min × 3) and visualized. Uninfected sera from the same pigs were used as a parallel negative-control. The negative-control experiment used the same method as mentioned above.

The scanned images of the Coomassie brilliant blue-stained 2-DE gels combined with the visualized western blot membranes were input to Image Master 2D Platinum 5.0 (GE) to identify species-specific spots.

### Proteins identification using MALDI-TOF/TOF-MS/MS analysis

The 2-DE gel spots corresponding to the *T. pseudospiralis* positive serum western blot spot were excised, and the proteins were digested in a gel with trypsin (Promega, Madison, USA). The samples mixed with an equivalent matrix solution (α-cyano-4-hydroxycinnamic acid) were applied for further MALDI-TOF/TOF-MS/MS analysis using a fuzzy logic feedback control system (Ultraflex III TOF/TOF mass spectrometer, Bruker, Karlsruhe, Germany). MS spectral data were acquired from the samples, and an automatically generated MS/MS list was further analyzed. All mass spectra were recorded in positive reflector mode and generated by accumulating data from 1000 laser shots. The following threshold criteria and settings were used: mass range of 800–4000 Da for detection, UV wavelength of 355 nm, laser frequency of 50 Hz, repetition rate of 200 Hz and accelerated voltage of 20,000 V. Peptide mass fingerprint (PMF) data were matched to the UniProt *Trichinella* and NCBInr *T. pseudospiralis* database using profound program under 50 ppm mass tolerance. Data were processed, and proteins were unambiguously identified searching against a comprehensive non-redundant sequence database by using the MASCOT software search engine (http://www.matrixscience.com). With the mascot search results, the protein probability score for the match, molecular weight (MW), isoelectric point (pI), number of peptide matches and percentage of the total sequence covered by the peptides were identified. Protein scores greater than 23 (*P* < 0.05) were considered significant.

### Bioinformatics analysis

Gene-ontology (GO) analysis was used to further uncover the molecular function and biological process of *T. pseudospiralis* ES proteins with the Quick Go Online Software (http://www.ebi.ac.uk/QuickGO/WebServices.html). *Trichinella pseudospiralis* ES proteins were divided into different clusters according to molecular function and biological process.

### Statistical analysis

All mass spectra data were analysed using Mascot software. Mascot adopts a probabilistic scoring algorithm for the identification of proteins, which was adapted from MOWSE algorithm. A *P* < 0.05 was considered as statistically significant.

## Results

### *Trichinella pseudospiralis* ES proteins analysis by 2-DE

**Fig. 1:**
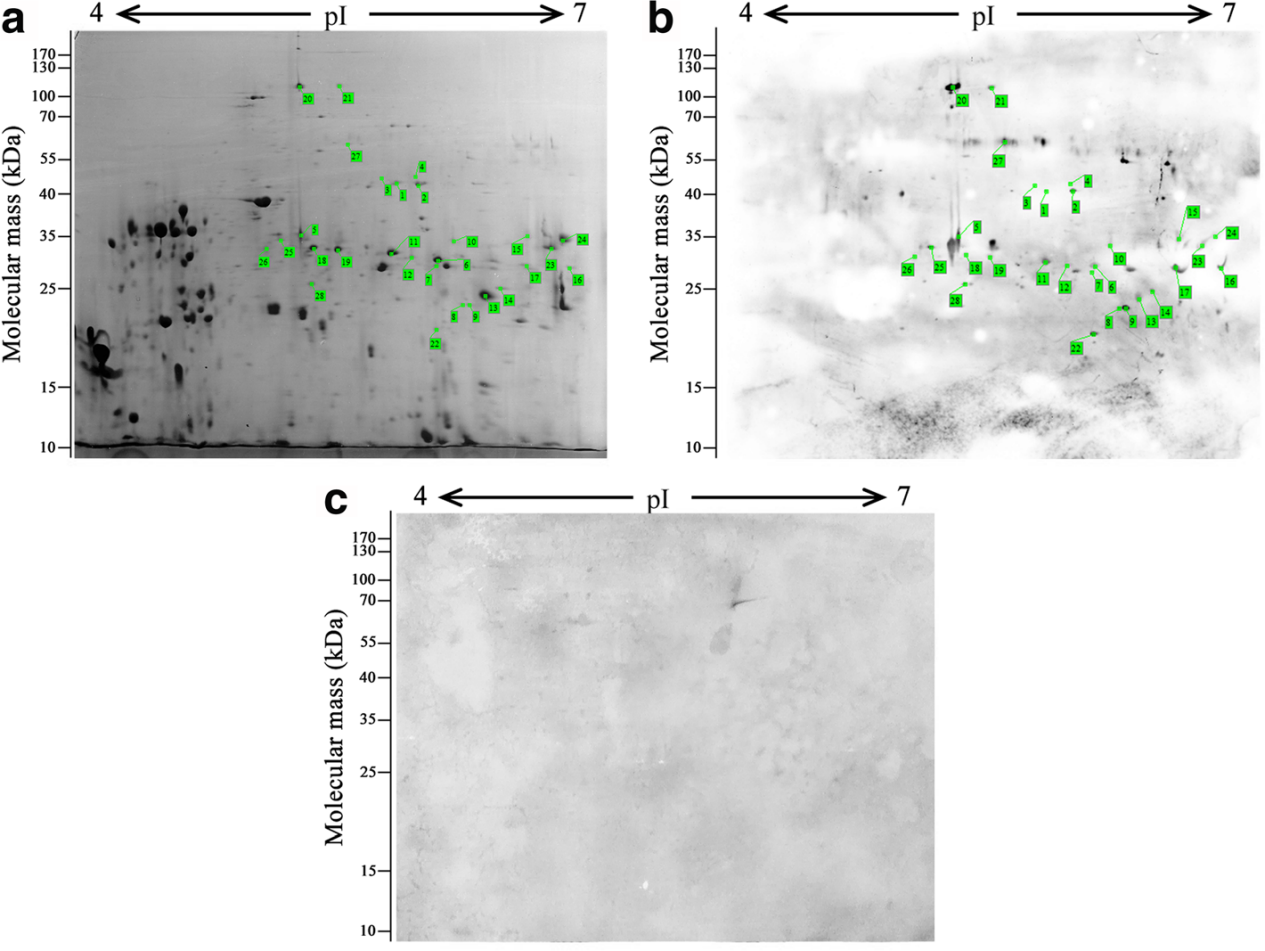
**a** Typical two-dimensional electrophoresis (2-DE) gel of *Trichinella pseudospiralis* adult worms excretory-secretory proteins separated in the first dimension in the pH range 4–7 and then in the second dimension on a 12% non-linear gradient polyacrylamide gel. The 2-DE gel was stained with coomassie brilliant blue G-250. Peptide spots selected for analysis are numbered. **b** Western blot of adult worms excretory-secretory proteins probed with pig infection sera at 26 days post-infection. Peptide spots selected for analysis are numbered. **c** Western blot of adult worms excretory-secretory probed with normal pig sera

In an attempt to identify species-specific parasite antigens, adult worms ES proteins were separated by 2-DE in a 17 cm, pH 4–7 IPG strip (preliminary experiments show that protein points mainly concentrated in the pH 4–7) and stained with Coomassie brilliant blue G250 (Fig. 1a). More than 400 spots were detected, with pI varying from 4 to 7 and MW from 10 to 170 kDa. Spots with a significant decrease (or increase) in their relative abundance were considered differentially expressed proteins if *P* < 0.05 and two spots had 1.5-fold differences in volume.

### Immunoblot analysis of ES proteins of adult worms and NBL of *T. pseudospiralis*

The results of the immunoblot of adult worms ES proteins were shown in Fig. 1b. Approximately 28 immunoreactive protein spots were identified by *T. pseudospiralis* 26 dpi positive serum and matched to the corresponding protein spot in Coomassie brilliant blue stained gels. These matched spots were named spot 1 to 28 and were selected to be further identified through matching of proteins by MALDI-TOF/TOF-MS/MS. These immunoreactive protein spots were observed to have MW ranging from 20 kDa to 130 kDa and pI values between 4 and 7. Most of these spots had observed MW ranging from 20 kDa to 40 kDa and pI values between 5 and 6. No proteins reacted to uninfected pig sera (Fig. 1c).

### Identification of *T. pseudospiralis* ES proteins by MALDI-TOF/TOF-MS/MS

Twenty-four of the 28 differentially expressed proteins were successfully identified by PMF, corresponding to 12 species-specific proteins. These proteins are listed in Table 1. Three criteria were used to identify species-specific proteins. First, several specific peptides for a given protein were found. Secondly, protein scores greater than 23 (*P* < 0.05) were considered significant. Finally, the observed MW and pI of the protein measured by 2-DE were in agreement with the calculated values. Spots 1, 12, 21, 23 and 27 were identified as adult-specific DNase II-10; spots 5, 7, 13, 16 and 26 were identified as poly-cysteine and histidine tailed protein isoform 2; spots 15, 17 and 24 were identified as serine protease; spots 3, 19 and 28 were identified as serine/threonine-protein kinase ULK3; other spots were identified as enolase, golgin-45, putative venom allergen 5, deoxyribonuclease II superfamily, chymotrypsin-like elastase family member 1, uncharacterized protein, peptidase inhibitor 16 and death-associated protein 1. Spots 4, 6, 11 and 14 were unmatched to any *T. spiralis* and *T. pseudospiralis* sequence currently in the database. Fewer peptide matches and a lower percent coverage were tolerated in making a putative assignment of ES proteins identity.

**Table 1:** Identification of *Trichinella pseudospiralis* excretory-secretory proteins by MALDI-TOF/TOF-MS/MS. All the MASCOT scores are >23 (*P* < 0.05)

No.	MS^a^	MP^b^	SC^c^	Mw(KDa)/pI^d^	Mw(KDa)/pI^e^	UniProt-NCBInr ID	Description
1	71	12	37	38.6/6.52	44.9/5.83	Q32R66_TRISP	Adult-specific DNase II-10
2	216	19	35	50.9/6.15	43.9/5.95	KRX96478.1	Enolase
3	83	11	13	110.6/9.40	47.2/5.74	KRY76695.1	Serine/threonine-protein kinase ULK3
4	–	–	–	–	–	–	Not identified
5	110	10	17	49.6/6.69	35.5/5.30	G4XSU5_TRISP	Poly-cysteine and histidine tailed protein isoform 2
6	–	–	–	–	–	–	Not identified
7	232	10	15	49.6/6.69	29.3/6.05	G4XSU5_TRISP	Poly-cysteine and histidine tailed protein isoform 2
8	37	1	2	50.7/9.20	23.1/6.19	E5SVR8_TRISP	Putative venom allergen 5
9	40	1	2	47.6/8.98	23.0/6.23	KRY01512.1	Chymotrypsin-like elastase family member 1
10	279	9	40	28.7/8.33	34.6/6.14	E5SZQ3_TRISP	Deoxyribonuclease II superfamily
11	–	–	–	–	–	–	Not identified
12	577	15	49	38.6/6.52	31.1/5.91	Q32R66_TRISP	Adult-specific DNase II-10
13	326	12	23	49.6/6.69	24.2/6.31	G4XSU5_TRISP	Poly-cysteine and histidine tailed protein isoform 2
14	–	–	–	–	–	–	Not identified
15	152	15	26	72.8/8.83	35.4/6.54	Q9BJM1_TRISP	Serine protease
16	266	13	23	49.6/6.69	28.9/6.77	G4XSU5_TRISP	Poly-cysteine and histidine tailed protein isoform 2
17	180	12	21	72.8/8.83	29.4/6.53	Q9BJM1_TRISP	Serine protease
18	79	11	32	41.5/8.45	33.0/5.37	KRZ27934.1	Golgin-45
19	80	12	10	110.6/9.40	32.7/5.50	KRY76695.1	Serine/threonine-protein kinase ULK3
20	66	10	42	30.9/9.20	122.2/5.29	KRY64535.1	Uncharacterized protein
21	225	12	35	38.6/6.52	122.6/5.51	Q32R66_TRISP	Adult-specific DNase II-10
22	331	8	16	57.1/7.56	20.1/6.05	KRY73124.1	Peptidase inhibitor 16, partial
23	606	19	61	38.6/6.52	33.0/6.67	Q32R66_TRISP	Adult-specific DNase II-10
24	124	20	30	72.8/8.83	34.9/6.73	Q9BJM1_TRISP	Serine protease
25	68	5	82	4.97/6.55	34.9/5.19	E5SL33_TRISP	Death-associated protein 1
26	318	12	21	49.6/6.69	32.9/5.12	G4XSU5_TRISP	Poly-cysteine and histidine tailed protein isoform 2
27	175	11	33	38.6/6.52	62.7/5.55	Q32R66_TRISP	Adult-specific DNase II-10
28	82	9	9	110.6/9.40	25.9/5.36	KRY76695.1	Serine/threonine-protein kinase ULK3

^a^Mascot score

^b^Matched peptide

^c^Sequence coverage (%)

^d^Theoretical

^e^Experimental

### Functional categories of ES proteins from adult worms and NBL of *T. pseudospiralis* by gene ontology

**Fig. 2:**
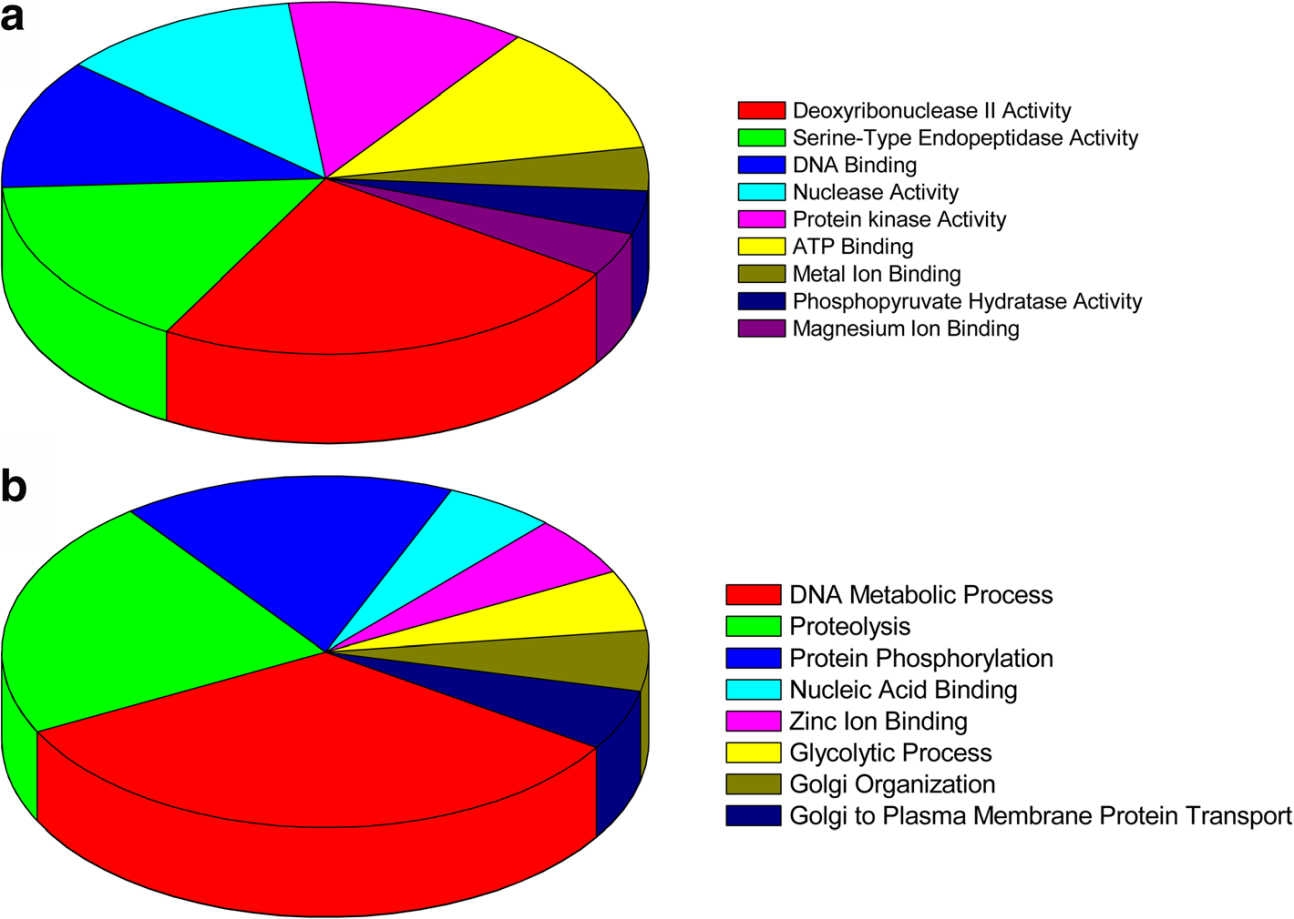
Gene ontology categories of proteins of adult worms excretory-secretory of *Trichinella pseudospiralis*. The identified proteins were classified into molecular function (**a**) and biological process (**b**) by Quick GO according to their gene oncology signatures

To further understand the functions of the ES proteins identified by early infection sera in this study, gene ontology annotation was performed. We utilize the UniProt database, and these 12 species-specific proteins were classified into a molecular function and biological process according to GO hierarchy (http://www.uniprot.org//). For the molecular function ontology, the proteins are related to peptidase activity acting on deoxyribonuclease II activity (GO: 0004531, 24%), serine-type endopeptidase activity (GO: 0004252, 16%), DNA binding (GO: 0003677, 12%), nuclease activity (GO: 0004518, 12%), protein kinase activity (GO: 0004672, 12%), ATP binding (GO:0005524, 12%), metal ion binding (GO: 0046872, 4%), phosphopyruvate hydratase activity (GO: 0004634, 4%) and magnesium ion binding (GO:0000287, 4%) (Fig. 2a). A large part of adult worms stage of ES proteins of *T. pseudospiralis* were related to DNA metabolic process (GO: 0006259, 33.33%), proteolysis (GO: 0006508, 22.22%), protein phosphorylation (GO: 0006468, 16.66%), nucleic acid binding (GO: 0003676, 5.56%), zinc ion binding (GO: 0008270, 5.56%), glycolytic process (GO: 0006096, 5.56%), Golgi organization (GO: 0007030, 5.56%) and Golgi to plasma membrane protein transport (GO: 0043001, 5.56%) (Fig. 2b).

## Discussion

Previous reports have been indicated that the ES products of parasites play important roles in development, adhesion, proteolysis and extracellular matrix organization of the organism [30]. During infection, the ES proteins may control the host immune reaction and recognition, acting as virulence factors or immune regulators [31]. Non-encapsulated species induce less inflammation than do encapsulated species [32], indicating that the ES proteins differ between these organisms. Thus, the study of identifying the ES proteins of adult worms and NBL of *T. pseudospiralis* are meaningful to reveal intestinal stage host-parasite interactions and understanding the phenomenon of less intestinal inflammation. Also, these ES proteins identified in our study might be candidates for use in developing early diagnostic tests and effective vaccines.

In this study, we successfully employed 2-DE and western blotting combined with MALDI-TOF/TOF-MS/MS to identify specific ES proteins of adult worms and NBL of *T. pseudospiralis*. Approximately 24 matched protein spots were recognized by PMF and were characterized to correlate with 12 different proteins. To further elucidate the functions of 12 different proteins, we utilized Quick GO online software, and these 12 proteins were categorized based on the GO annotation of biological process and molecular functions. Most of the molecular functions of ES proteins were DNase II activity and serine-type endopeptidase activity. Several proteins had multiple spots with different pI and MW, such as adult-specific DNase II-10, poly-cysteine and histidine tailed protein isoform 2, serine/threonine-protein kinase ULK3 and serine protease. These proteins might undergo alternative splicing, post-translational modifications, binding to different co-factors or protein processing [17, 33], possibly involving biological regulation, glycolytic process, metabolic process, protein folding and proteolysis.

Previous studies have indicated that the parasitic ES products are composed of many functional proteins involved in host-parasite interactions. In this study, many functional proteins were also identified, especially multiple isoforms of DNase II and serine proteases. DNase II and serine proteases have been proved critical for the invasion of the host and modulating host immune responses [34]. DNase II is a well-known acidic endonuclease that fulfils a variety of functions from degrading DNA associated with apoptosis and dietary DNA to modulating host immune responses. Multiple isoforms of the DNase II identified in *T. pseudospiralis* ES products suggest that DNase II may function as self-protective molecules by alleviating host intestinal inflammation by cleaving DNA from apoptotic host cells during the larvae invasion of epithelial cells. Furthermore, a novel microbicidal mechanism beyond cell death, recently described as extracellular traps (ETs), was identified in many innate effector cells. ETs are weblike structures composed of chromatin and granular and cytoplasmic proteins, which ensnare and kill microorganisms including bacteria, fungi and parasites [35–37]. However, some of the microorganisms such as *Staphylococcus aureus*, *Streptococcus pneumonia*, *Vibrio cholera* and *Leishmania amazonensis* have been proved to express endonucleases that efficiently degrade DNA filaments from ETs, allowing these microorganisms to escape the toxic effects of ETs and to invade or spread throughout the host [38–41]. The DNase II secreted from the *T. pseudospiralis* may have the similar function to escape the fatal attraction of ETs.

Serine proteases reportedly are expressed at different stages and have different functions in establishing the parasitism of *T. spiralis* [42]. Indeed, serine proteinases partially purified from the ES products of *T. spiralis* adult worms exhibit strong biological activity, which plays a vital role in the degradation of intestinal tissues and helps parasites penetrate a diverse range of host tissue barriers, acquire nutrients and evade the host immune response [43]. Meanwhile, serine proteases are also involved in mediating apoptosis-like cell death and phagocytosis [44], which may contribute to parasite-induced immunosuppression. In this study, multiple serine proteases including chymotrypsin-like elastase were identified by sera at 26 dpi of *T. pseudospiralis*, which might be correlative with the invasion of host enterocytes and the suppression of inflammation. However, the functions of serine protease families are strongly context-dependent in parasite infection, and further experimental analyses are necessary to improve the reliability of the functional interpretation of our results.

Compared with the ES products of *T. pseudospiralis* ML, many stage-specific molecules, such as adult-specific DNase II-10, poly-cysteine and histidine tailed protein isoform2, serine protease, serine/threonine-protein kinase ULK3, enolase, putative venom allergen 5, chymotrypsin-like elastase family member 1, uncharacterized protein, peptidase inhibitor 16, death-associated protein 1, deoxyribonuclease II superfamily and golgin-45 were identified in adult worms. Apart from DNAse II and serine protease families, the poly-cysteine and histidine tailed protein (PCHTP) were also identified in several spots. The PCHTP as a metalloprotein is a new nematode ES-specific protein family of PCHTP-Poly-cysteine proteins, which are unique to order *Trichocephalida* [45, 46]. In previous studies, PCHTP sequence analysis showed typical metal binding residues, suggesting that multiple potential metal binding sites may be formed [47, 48]. These metal binding sites consist of cysteine-rich and poly-histidine regions that can bind different bivalent metal ions such as Fe, Ni, Cu, Co and Zn and for the first time have been identified in the ES products of non-encapsulated larvae, which most likely play a role in transporting or storing metal ions [45, 46]. Another three proteins, namely, golgin-45, venom allergen 5 and enolase, were also identified in this study. Golgin-45 is located on the surface of the medial cisternae of the Golgi complex and maintains the structure of Golgi and secretory protein transport [49]. The venom allergen five gene is identified in many metazoans as the major allergen and is often associated with allergic responses in humans [50, 51]. The venom allergen five proteins are part of a cocktail of salivary proteins believed to function either in suppressing the host immune system or preventing clotting to prolong feeding [52]. The putative venom allergen five secreted by *T. pseudospiralis* may help adult worms and NBL evade the host immune response. Enolase is a multifunctional protein that catalyzes the reversible dehydration of 2-phospho-D-glycerate (2PGA) to phosphoenolpyruvate (PEP). Parasitic enolase can enhance the activation of plasminogen [53]. Therefore, as a component of ES products, enolase has been confirmed to be a very important virulence factor during invasion into the host [54]. Furthermore, Wang X et al. recently reported invasion, immune evasion and pathogenesis mechanisms through which *Clonorchis sinensis* enolase (*Csenolase*) from ES products participating in plasminogen acquisition and proteolysis may enhance extracellular matrix degradation and control the parasite growth [55]. As a potential vaccine, *Csenolase* has shown high immunogenicity and has exhibited considerable protective efficacy [56, 57]. In the same way, enolase from *T. pseudospiralis* may contribute to the tissue migration of NBL and host-parasite interactions and may be a vaccine candidate or diagnostic protein.

## Conclusions

In summary, 2-DE and western blotting combined with MALDI-TOF/TOF-MS/MS was used to screen the early candidate antigens from the ES proteins of *T. pseudospiralis* adult worms in this study. Twelve different proteins including adult-specific DNase II-10, poly-cysteine and histidine tailed protein isoform2, serine protease, serine/threonine-protein kinase ULK3, enolase, putative venom allergen 5, chymotrypsin-like elastase family member 1, uncharacterized protein, peptidase inhibitor 16, death-associated protein 1, deoxyribonuclease II superfamily and golgin-45 stage-specific proteins were identified in total. The identification of ES products is critical to understanding the host-parasite interaction and may have broader implications for research on the mechanisms of immunosuppression.

## Abbreviations

2-DE: two-dimensional gel electrophoresis
2PGA: 2-phospho-D-glycerate
Csenolase: *Clonorchis sinensis* enolase
DNase II: deoxyribonuclease II
dpi: days post-infection
ELISA: enzyme-linked immunosorbent assay
ES: excretory-secretory
GO: gene-ontology
IEF: isoelectric focusing
IPG: immobilized pH gradient
MALDI-TOF/TOF-MS/MS: matrix-assisted laser desorption/ionization time of flight mass spectrometry
ML: muscle larvae
MW: molecular weight
NBL: newborn larvae
PCHTP: poly-cysteine and histidine tailed protein
PEP: phosphoenolpyruvate
pI: isoelectric point
PMF: peptide mass fingerprint
PVDF: polyvinylidene fluoride
SDS: sodium dodecyl sulfate
TEMED: tetramethylethylenediamine

## References

[1] Dupouy-Camet J. Presidential address of ICT12 conference: "*Trichinella* and trichinellosis - a never ending story". Vet Parasitol. 2009;159(3–4):194–6. 10.1016/j.vetpar.2008.10.064 19054620

[2] Pozio E, Hoberg E, La Rosa G, Zarlenga DS (2009). Molecular taxonomy, phylogeny and biogeography of nematodes belonging to the *Trichinella* genus. Infect Genet Evol.

[3] Dupouy-Camet J (2006). Trichinellosis: still a concern for Europe. Euro Surveill.

[4] Pozio E, Zarlenga DS (2013). New pieces of the *Trichinella* puzzle. Int J Parasitol.

[5] Li CK, Chung YY, Ko RC (1999). The distribution of excretory/secretory antigens during the muscle phase of *Trichinella spiralis* and *T. pseudospiralis* infections. Parasitol Res.

[6] Hurnikova Z, Snabel V, Pozio E, Reiterova K, Hrckova G, Halasova D (2005). First record of *Trichinella pseudospiralis* in the Slovak Republic found in domestic focus. Vet Parasitol.

[7] Szell Z, Marucci G, Ludovisi A, Gomez-Morales MA, Sreter T, Pozio E (2012). Spatial distribution of *Trichinella britovi*, *T. spiralis* and *T. pseudospiralis* of domestic pigs and wild boars (*Sus scrofa*) in Hungary. Vet Parasitol.

[8] Pozio E, Christensson D, Steen M, Marucci G, La Rosa G, Brojer C (2004). *Trichinella pseudospiralis* foci in Sweden. Vet Parasitol.

[9] Szell Z, Marucci G, Bajmoczy E, Cseplo A, Pozio E, Sreter T (2008). Spatial distribution of *Trichinella britovi*, *T. pseudospiralis* and *T. spiralis* in red foxes (*Vulpes vulpes*) in Hungary. Vet Parasitol.

[10] Andrews JR, Ainsworth R, Abernethy D (1993). *Trichinella pseudospiralis* in man. Lancet.

[11] Pozio E, Shaikenov B, La Rosa G, Obendorf DL (1992). Allozymic and biological characters of *Trichinella pseudospiralis* isolates from free-ranging animals. J Parasitol.

[12] Kehayov I, Tankov C, Komandarev S, Kyurkchiev S (1991). Antigenic differences between *Trichinella spiralis* and *T. pseudospiralis* detected by monoclonal antibodies. Parasitol Res.

[13] Kuratli S, Lindh JG, Gottstein B, Smith DF, Connolly B (1999). *Trichinella* spp.: differential expression of two genes in the muscle larva of encapsulating and nonencapsulating species. Exp Parasitol.

[14] Vassilatis DK, Despommier DD, Polvere RI, Gold AM, Van der Ploeg LH (1996). *Trichinella pseudospiralis* secretes a protein related to the *Trichinella spiralis* 43-kDa glycoprotein. Mol Biochem Parasit..

[15] Wu Z, Nagano I, Takahashi Y (1998). Differences and similarities between *Trichinella spiralis* and *T. pseudospiralis* in morphology of stichocyte granules, peptide maps of excretory and secretory (E-S) products and messenger RNA of stichosomal glycoproteins. Parasitology.

[16] Bolas-Fernandez F, Corral Bezara LD (2006). TSL-1 antigens of *Trichinella*: an overview of their potential role in parasite invasion, survival and serodiagnosis of trichinellosis. Res Vet Sci.

[17] Robinson MW, Greig R, Beattie KA, Lamont DJ, Connolly B (2007). Comparative analysis of the excretory-secretory proteome of the muscle larva of *Trichinella pseudospiralis* and *Trichinella spiralis*. Int J Parasitol.

[18] Ko RC, Fan L (1996). Heat shock response of *Trichinella spiralis* and *T. pseudospiralis*. Parasitology.

[19] Mak CH, Ko RC (1999). Characterization of endonuclease activity from excretory/secretory products of a parasitic nematode. Trichinella spiralis Eur J Biochem.

[20] Moczon T, Wranicz M (1999). *Trichinella spiralis*: proteinases in the larvae. Parasitol Res.

[21] Arden SR, Smith AM, Booth MJ, Tweedie S, Gounaris K, Selkirk ME (1997). Identification of serine/threonine protein kinases secreted by *Trichinella spiralis* infective larvae. Mol Biochem Parasit..

[22] Gounaris K, Thomas S, Najarro P, Selkirk ME (2001). Secreted variant of nucleoside diphosphate kinase from the intracellular parasitic nematode *Trichinella spiralis*. Infect Immun.

[23] Nagano I, Wu Z, Nakada T, Matsuo A, Takahashi Y (2001). Molecular cloning and characterization of a serine proteinase inhibitor from *Trichinella spiralis*. Parasitology.

[24] Mak CH, Ko RC (2001). DNA-binding activity in the excretory-secretory products of *Trichinella pseudospiralis* (Nematoda: Trichinelloidea). Parasitology.

[25] Gounaris K (2002). Nucleotidase cascades are catalyzed by secreted proteins of the parasitic nematode *Trichinella spiralis*. Infect Immun.

[26] Cuttell L, Gomez-Morales MA, Cookson B, Adams PJ, Reid SA, Vanderlinde PB (2014). Evaluation of ELISA coupled with western blot as a surveillance tool for *Trichinella* infection in wild boar (*Sus scrofa*). Vet Parasitol.

[27] Nockler K, Reckinger S, Broglia A, Mayer-Scholl A, Bahn P (2009). Evaluation of a western blot and ELISA for the detection of anti-*Trichinella*-IgG in pig sera. Vet Parasitol.

[28] Liu MY, Wang XL, Fu BQ, Li CY, Wu XP, Le Rhun D (2007). Identification of stage-specifically expressed genes of *Trichinella spiralis* by suppression subtractive hybridization. Parasitology.

[29] Bradford MM (1976). A rapid and sensitive method for the quantitation of microgram quantities of protein utilizing the principle of protein-dye binding. Anal Biochem.

[30] Maizels RM, Yazdanbakhsh M (2003). Immune regulation by helminth parasites: cellular and molecular mechanisms. Nat Rev Immunol.

[31] Gahoi S, Gautam B (2017). Genome-wide analysis of excretory/secretory proteins in root-knot nematode, *Meloidogyne incognita* provides potential targets for parasite control. Comput Biol Chem.

[32] Bruschi F, Marucci G, Pozio E, Masetti M (2009). Evaluation of inflammatory responses against muscle larvae of different *Trichinella* species by an image analysis system. Vet Parasitol.

[33] Bien J, Nareaho A, Varmanen P, Gozdzik K, Moskwa B, Cabaj W (2012). Comparative analysis of excretory-secretory antigens of *Trichinella spiralis* and *Trichinella britovi* muscle larvae by two-dimensional difference gel electrophoresis and immunoblotting. Proteome Sci.

[34] Liu RD, Jiang P, Wen H, Duan JY, Wang LA, Li JF (2016). Screening and characterization of early diagnostic antigens in excretory-secretory proteins from *Trichinella spiralis* intestinal infective larvae by immunoproteomics. Parasitol Res.

[35] Behrendt JH, Ruiz A, Zahner H, Taubert A, Hermosilla C (2010). Neutrophil extracellular trap formation as innate immune reactions against the apicomplexan parasite *Eimeria bovis*. Vet Immunol Immun.

[36] Brinkmann V, Reichard U, Goosmann C, Fauler B, Uhlemann Y, Weiss DS (2004). Neutrophil extracellular traps kill bacteria. Science.

[37] Urban CF, Reichard U, Brinkmann V, Zychlinsky A (2006). Neutrophil extracellular traps capture and kill *Candida albicans* yeast and hyphal forms. Cell Microbiol.

[38] Beiter K, Wartha F, Albiger B, Normark S, Zychlinsky A, Henriques-Normark B (2006). An endonuclease allows *Streptococcus pneumoniae* to escape from neutrophil extracellular traps. Curr Biol.

[39] Berends ET, Horswill AR, Haste NM, Monestier M, Nizet V, von Kockritz-Blickwede M (2010). Nuclease expression by *Staphylococcus aureus* facilitates escape from neutrophil extracellular traps. J Innate Immun.

[40] Guimaraes-Costa AB, DeSouza-Vieira TS, Paletta-Silva R, Freitas-Mesquita AL, Meyer-Fernandes JR, Saraiva EM (2014). 3′-nucleotidase/nuclease activity allows *Leishmania* parasites to escape killing by neutrophil extracellular traps. Infect Immun.

[41] Seper A, Hosseinzadeh A, Gorkiewicz G, Lichtenegger S, Roier S, Leitner DR (2013). *Vibrio cholerae* evades neutrophil extracellular traps by the activity of two extracellular nucleases. PLoS Pathog.

[42] Yang Y, Wen Y, Cai YN, Vallee I, Boireau P, Liu MY (2015). Serine proteases of parasitic helminths. Korean J Parasitol.

[43] Todorova VK, Stoyanov DI (2000). Partial characterization of serine proteinases secreted by adult *Trichinella spiralis*. Parasitol Res.

[44] Egger L, Schneider J, Rheme C, Tapernoux M, Hacki J, Borner C (2003). Serine proteases mediate apoptosis-like cell death and phagocytosis under caspase-inhibiting conditions. Cell Death Differ.

[45] Radoslavov G, Jordanova R, Teofanova D, Georgieva K, Hristov P, Salomone-Stagni M (2010). A novel secretory poly-cysteine and histidine-tailed metalloprotein (Ts-PCHTP) from *Trichinella spiralis* (Nematoda). PLoS One.

[46] Sbirkova HI, Radoslavov GA, Hristov PI, Shivachev BL (2013). Crystallographic conditions of the heterologically expressed recombinant metal-binding protein Ts-PCHTP. Bulg Chem Commun.

[47] Gregory DS, Martin AC, Cheetham JC, Rees AR (1993). The prediction and characterization of metal binding sites in proteins. Protein Eng.

[48] Yamashita MM, Wesson L, Eisenman G, Eisenberg D. Where metal ions bind in proteins. Proc Natl Acad Sci USA. 1990;87(15):5648–52. 10.1073/pnas.87.15.5648 PMC54384 2377604

[49] Short B, Preisinger C, Korner R, Kopajtich R, Byron O, Barr FA (2001). A GRASP55-rab2 effector complex linking Golgi structure to membrane traffic. J Cell Biol.

[50] King TP, Spangfort MD (2000). Structure and biology of stinging insect venom allergens. Int Arch Allergy Imm.

[51] Muller UR, Johansen N, Petersen AB, Fromberg-Nielsen J, Haeberli G (2009). *Hymenoptera* venom allergy: analysis of double positivity to honey bee and *Vespula* venom by estimation of IgE antibodies to species-specific major allergens Api m1 and Ves v5. Allergy.

[52] Ribeiro JM, Francischetti IM (2003). Role of arthropod saliva in blood feeding. Sialome and post-sialome perspectives. Annu Rev Entomol.

[53] Nakada T, Nagano I, Wu Z, Takahashi Y (2005). Molecular cloning and functional expression of enolase from *Trichinella spiralis*. Parasitol Res.

[54] Antunez K, Anido M, Arredondo D, Evans JD, Zunino P (2011). Paenibacillus larvae enolase as a virulence factor in honeybee larvae infection. Vet Microbiol.

[55] Wang X, Chen W, Hu F, Deng C, Zhou C, Lv X (2011). *Clonorchis sinensis* enolase: identification and biochemical characterization of a glycolytic enzyme from excretory/secretory products. Mol Biochem Parasit.

[56] Wang X, Chen W, Tian Y, Mao Q, Lv X, Shang M (2014). Surface display of *Clonorchis sinensis* enolase on *Bacillus subtilis* spores potentializes an oral vaccine candidate. Vaccine.

[57] Wang X, Hu F, Hu X, Chen W, Huang Y, Yu X (2014). Proteomic identification of potential *Clonorchis sinensis* excretory/secretory products capable of binding and activating human hepatic stellate cells. Parasitol Res.

